# Environmental Sustainability Framework for Plastic Waste Management—a Case Study of Bubble Tea Industry in Malaysia

**DOI:** 10.1007/s41660-022-00230-w

**Published:** 2022-02-16

**Authors:** Chie Jian Lee, Lam Chang, Jully Tan

**Affiliations:** School of Engineering, Monash University Malaysia, Jalan Lagoon Selatan, 47500 Subang Jaya, Bandar Sunway, Selangor Malaysia

**Keywords:** Plastic waste, Sanitary landfill, Waste to energy incineration, Life cycle assessment, Sustainability framework

## Abstract

Economic growth and rapid industrialisation have led to enormous increase in municipal solid waste (MSW). Lack of waste management alternatives and ineffective waste policy implementation are the major challenges for government to materialise a sustainable solid waste management framework, especially for plastic waste. Booming of the food and beverage (F&B) industry has aggravated the situation by generating more plastic waste with no economic values. Hence, this study aims to evaluate the overall environmental performance of existing and alternative waste management technologies that are available in Malaysia based on net greenhouse gas (GHG) emission in terms of carbon dioxide equivalent (kg CO_2_-eq) per tonne of plastic waste that are analysed through life cycle assessment (LCA) methodology. LCA result has proven that Scenario B (waste to energy (WTE) incineration) is more environmentally preferable as it had a negative net GHG emission of − 573.80 kg CO_2_-eq as compared to GHG emission of existing Scenario A (sanitary landfill) of 566.15 kg CO_2_-eq. Negative net GHG emission in WTE incineration was mainly due to higher GHG saving achieved through cleaner electricity generation as compared to conventional power production. This alternative technology was proven to have the potential to reduce the dependence on landfills and is served as the basis of environmental sustainability framework development for plastic waste management based on case study in Malaysia. This framework can be served as the baseline for the local authorities or policy makers for other plastic waste generation hotspots other than bubble tea industry to improve plastic waste management via WTE incineration.

## Introduction

Recently, the bubble tea industry, a sector in the food and beverage (F&B) industry, has created a hype to the whole world with their signature brown sugar milk bubble tea and since then, bubble tea has become an iconic drink in which every youngster is craving for. Besides, bubble tea shops can be found almost everywhere around commercial areas due to the hype, especially at the famous Bubble Tea Streets in Subang Jaya, Malaysia. It is reported that there are approximately 74 brands of bubble tea vendors in Malaysia (Tan 2019). However, high demand of bubble tea has led to significant increase in production of plastic waste.

Approximately 242 million tonnes of global plastic waste has been generated, which was 12% of the global municipal solid waste in accordance to the statistics from The World Bank in 2016 (Kaza et al. 2018). In addition, 91% of the plastic waste is not recyclable. Due to its non-biodegradable characteristic, plastic waste leads to many environmental impacts such as abiotic resource depletion, global warming potential, acidification, eutrophication, and human toxicity (Chang and Tan 2021; Moy et al. 2021) as they are either being released to the open environment as litter or being disposed into landfills, and only 12% of the plastic waste has undergone proper treatment such as incineration. The food and beverage (F&B) industry is the primary source of plastic waste generation and approximately 141 megatonnes of global plastic waste has been generated by this industry in 2015. This is because packaging plastic products used by this industry have a very short life cycle of not more than 6 months. Therefore, this industry is responsible for two-thirds of the global plastic waste generation (Geyer et al. 2017; Ncube et al. 2021).

According to current Solid Waste Management Lab Report produced by the Ministry of Housing and Local Government Malaysia (KPKT) (2015), landfill is the only existing waste disposal method for plastic waste in Malaysia and plastic waste takes longer time to degrade in landfill as compared to other types of municipal solid waste (MSW). Besides, the government had set a target to divert 40% waste from landfills by year 2020 (KPKT 2015). However, the booming of the bubble tea industry in 2019 has aggravated the situation making the target harder to achieve. This is because bubble tea vendors tend to utilise petroleum-based single-use plastics with added additives as packaging materials in which are either non-recyclable or rejected by recycling facilities as they can contaminate batches of recycled plastic and harm recycling infrastructure.

The national solid waste management policy (NSWMP) is the current waste management policy in Malaysia that deals with plastic waste issue. The goals of this policy are (i) to construct a solid waste management framework which is sustainable, socially acceptable, and cost-effective and (ii) to introduce concept of reduce, reuse, and recycling in implementation of solid waste management system that is based on the hierarchy of solid waste. However, this waste management policy is ineffective in materialising a sustainable solid waste management framework that deals with increase in plastic waste issue due to a number of reasons. Firstly, lack of political will in implementation of the policy via formal endorsement is the main root cause. Besides, there is lack of economic incentives for recycling activities and funding for implementation of advanced technological solutions other than LGRS which leads to ineffective policy implementation (Wee et al. 2017). Moreover, lack of recycling awareness among Malaysians has led to low rate of plastic recycling (Alias et al. 2018). Furthermore, lack of expertise and technology to practice plastic waste management effectively is also one of the reasons. A first technological solution, the waste to energy (WTE) incinerator in Malaysia is expected to begin its operation in late 2021 (Aziz 2020). As a result, high rates of plastic waste generation from the bubble tea industry will speed up the filling up rate of landfill in the long run. Therefore, all these factors have hastened the government’s plan to begin operation of the WTE incinerator as soon as possible to tackle the increasing plastic waste issue.

It is also well noting that only 10 out of the 166 landfills in Malaysia are sanitary landfills with landfill gas recovery system (LGRS) in which 94% of the landfills release methane (CH_4_) to the environment directly without capturing it for electricity generation which leads to serious global warming issue (KPKT 2015). As Malaysia has yet to begin operation of any WTE incinerator, therefore the environmental performance of this alternative technology is not known. As a result, GHG emission from sanitary landfill and WTE incineration cannot be compared which hinders the sustainable plastic waste management framework development for government to tackle high rates of plastic waste generation issue.

Plastic packaging contributes as the largest end-use market segment that accounts almost 40% of the total worldwide plastic usage (Akenji et al. 2020). A report shows that the F&B industry is under huge pressure to allay the concern regarding global warming as this industry is responsible for 21 to 37% of the world’s manmade GHG emission (Wheeler 2020). This statistic can be self-explained in our daily life where the packaging materials for many F&B items are made of plastics such as processed food from grocery store and takeaway food from restaurants or coffee shops. It takes time for the F&B industry in the whole world to fully substitute the plastic packaging with other materials in order to reduce the global warming impact caused by disposing the plastic waste. In the meantime, developing and third world nations will continue to face challenges to find a sustainable solution to replace landfilling in order to address high rates of plastic waste generation from the F&B industry.

In the case of large amounts of solid waste, WTE incineration is the more widely known and less expensive option when compared to other more sophisticated thermal waste management technology. As a result, the purpose of the study is to assess the overall environmental performance of existing sanitary landfills and alternative WTE incineration in Malaysia using life cycle assessment (LCA). The waste management of the bubble tea industry was chosen because it is a hotspot for plastic waste generation in the recent F&B industry. Furthermore, the study’s goal is to create an environmental sustainability framework for plastic waste management based on a case study that highlights the engagement of government, waste contractors, vendors, and the public to ensure the proposed framework’s long-term application in efficiently managing municipal plastic waste. The work flow was arranged such that the methodology of the LCA was detailed, followed by the results and discussion. Finally, the conclusion and recommendations are presented.

## Literature Review

### Landfilling in Malaysia

A sanitary landfill is a well-designed system that has geomembrane which is made of high-density polyethylene (HDPE) as foundation to prevent underground leaking, a drainage system to channel leachate accumulated at the bottom of landfill to leachate treatment plant and LGRS to capture methane (CH_4_). The dominant types of GHG emission from landfilling are CH_4_ and CO_2_. Landfilling is the major source of CH_4_ emission which accounts for 53% of CH_4_ emission in Malaysia and CH_4_ emission from non-sanitary landfills is much greater than the emission from sanitary landfills. It is predicted that 370,000 tonnes of CH_4_ is to be produced in 2020, which is equivalent to 9.25 million tonne CO_2_-eq (Yong et al. 2019). This amount of greenhouse CH_4_ is 28 times more potent than CO_2_ in warming the earth. Although sanitary landfills can reduce these negative environmental impacts as compared to non-sanitary landfills, establishing a new sanitary landfill to trap CH_4_ can be a challenging task for authorities due to land scarcity, high land cost around urban area, and negative perception from residents/communities who are living near the landfills.

### Incineration in Malaysia

Incineration is a thermal waste treatment technology that converts waste into ash, heat, and flue gas under high temperature. The dominant types of GHG emission from incineration are CO_2_ and nitrous oxide (N_2_O). There are two types of incineration, i.e., WTE and conventional incineration, in which the latter one does not harness heat energy from flue gas to generate electricity. Incineration is the most common thermal treatment of plastic waste as compared to pyrolysis and gasification. There are several major challenges for Malaysia to establish WTE incinerators. Firstly, the moisture content in the municipal solid waste in Malaysia that is approximately 45% will lead to ineffective incineration process. However, non-organic wastes such as plastic waste with very low moisture content and high calorific value make it suitable for thermal treatment (Yong et al. 2019). Secondly, high capital and maintenance costs hinder developing countries like Malaysia to invest money on establishing more WTE incinerators. Nevertheless, incineration saves cost in the long run as it reduces volume of waste by converting them into energy.

The most common type of WTE incineration is the moving grate incinerator which has numerous benefits such as it does not require waste sorting or pre-treatment of waste, accommodates large waste volume, and has the ability to handle all types of MSW as well as able to achieve complete combustion to maximise the extraction of heat energy from waste for electricity generation or district heating (Lew 2020). Malaysia has installed its first large-scale WTE incinerator in Negeri Sembilan. This incinerator is scheduled to operate in 2021 which can help to divert MSW including plastic waste from landfills (Aziz 2020). Tan et al. (Tan et al. 2015) conducted a review on the WTE technologies to compare their advantages and disadvantages, which include the environmental assessment. The air pollution control system has undergone development in recent years to effectively capture harmful pollutants released from incineration such as dioxins, furans, mercury, and polychlorinated biphenyl (particulate matter). WTE incinerators in Japan utilise adsorption on the activated carbon method followed by filtration by bag filters or injection of thiourea without manipulating incineration conditions to effectively remove dioxins, furans, and mercury. Besides, combination of air pollution control technologies such as electrostatic precipitators, cyclones, and baghouse filters is very effective in removing polychlorinated biphenyls (CCET guideline series on intermediate municipal solid waste treatment technologies: waste-to-energy incineration 2020).

### WTE Incineration—a Long-Term Solution for Plastic Waste Management

Recycling plastic waste has its challenges as majority of plastic products fall under the category of plastic grades 4 to 7 which are rarely to be recycled or non-recyclable (Kaza et al. 2018). Plastics of all grades usually contain additives, adding difficulties in recycling. Moreover, plastic products such as packaging plastics often consist of more than a single type of plastic grade, which are usually termed as mixed plastics, adding more difficulties in sorting and recycling these plastics. Besides this, production of biodegradable plastics is very low, which is only 1% of the global plastic production. Plastic products from grades 1 and 2 which are widely recycled can only be recycled once or twice and recycling processes often produce plastic products of lower quality and these products will eventually be ended up either in the landfills or incinerators (Ritchie and Roser 2018). Thus, it is more economical to incinerate plastic waste if these plastics are too difficult to be sorted and recycled. Furthermore, recycling plastic is difficult as compared to recycling metals and glasses. Therefore, many developed nations get rid of their plastic waste in a cheap way by exporting them to developing nations such as China and Malaysia for recycling purposes (Wang et al. 2019). However, many of these discarded plastics are not recyclable and this plastic waste will eventually be ended up in landfills or incinerators in which both waste management technologies can create electricity from the plastic waste. Although recycling plastic waste is a better option in accordance to waste hierarchy, WTE technology is compatible or even does better than recycling in circular economy from overall sustainability point of view as it recovers both energy and materials from non-recyclable waste which keeps the environment and humans free from toxic substances (Caneghem et al. 2019).

New Zealand (NZ), a country having a very similar waste composition as Malaysia, is keen to explore sustainable waste treatment alternatives such as WTE technologies to divert MSW from landfills. Three WTE thermal treatment technologies were studied and compared based on contexts of NZ, which are incineration, pyrolysis, and gasification. The result of the literature suggested that incineration is still the most suitable WTE technology to replace landfill due to its ability to largely reduce volume of waste, matured technology, ability to handle variant of MSW, and suitable for large-scale application to deal with increasing MSW in the long run. Besides, pyrolysis and gasification create more side products that require further downstream treatment processes which will lead to greater emission whereas bottom ash from incineration can be used as a construction material. Moreover, modern WTE incinerators have an electrical energy production efficiency of 30% which is higher than the efficiency of gasification and pyrolysis of 27% and 25%, respectively (Perrot and Subiantoro 2018).

According to the United States Environmental Protection Agency (USEPA), incineration of MSW generates lesser GHGs than coal, oil, and natural gas as shown in Table 1 (USEPA 2016). This is good news for Malaysians as 86% of the current power generation in Malaysia are from non-renewable resources as shown in Table 2 (Abdullah et al. 2019). Therefore, WTE incineration serves as an alternative option to reduce dependence on fossil fuels, divert 40% of MSW, especially plastic waste from landfills, and reduce effect of global warming by reducing GHG emission from burning of fossil fuels and landfilling.

**Table 1:** Summary of CO_2_ emission from combustion of different types of combustible fuel (Perrot and Subiantoro 2018)

Fuel	GHG emission (kg CO_2_ per MWh)
MSW	461
Coal	1020
Oil	758
Natural gas	515

**Table 2:** Power generation sources and respective contribution to total energy production of Malaysia in 2016 (USEPA 2016)

Power generation sources	Contribution to energy production of Malaysia in percentage (%)
Natural gas	43.5
Coal	42.5
Hydropower	13.0
Oil	0.3
Diesel	0.3
Others	0.4

## Methodology

The method of this study is LCA which focuses on utilisation of plastic cups and downstream management of plastic waste from a designated Bubble Tea Street in Subang Jaya, Malaysia. LCA has four main components, which are goal and scope definition, inventory analysis, impact assessment, and interpretation of result (Arvanitoyannis 2008). LCA enables ones to perform different analyses based on defined system boundary, inventory analysis, and made assumptions (Tan et al. 2017).

Three important inputs for this LCA study are (i) plastic cup grades, (ii) number of bubble tea shops, and (iii) type of electrical appliances used in bubble tea shops via site visit. Plastic cup grades used by each vendor can be known by taking pictures of the cups via site visit. The number of bubble tea establishments is counted using Google Maps, while the sort of electrical appliances used to make bubble milk tea is determined by interviewing bubble tea merchants.

Assumptions of this LCA study are as follows:

1. Only CO_2_ is considered for combustion of fossil fuel for transportation and electricity generation as emission factors of CH_4_ and N_2_O is much smaller than CO_2_; typically both GHGs contribute to only 1% of the overall GHG emission from the combustion process (Eggleston et al. 2006).
2. The WTE incinerator besides Jeram Sanitary Landfill is estimated to be ready by year 2023 but the actual commencement date is unknown (Zainul 2018).
3. Volume of landfill gas is roughly 50% CO_2_ and 50% CH_4_ (USEPA 2020).
4. CH_4_ is excluded from the LCA study as it typically contributes to less than 0.01% of total GHG emission from incineration (Eggleston et al. 2006).

### Goal and Scope Definition

**Fig. 1:**
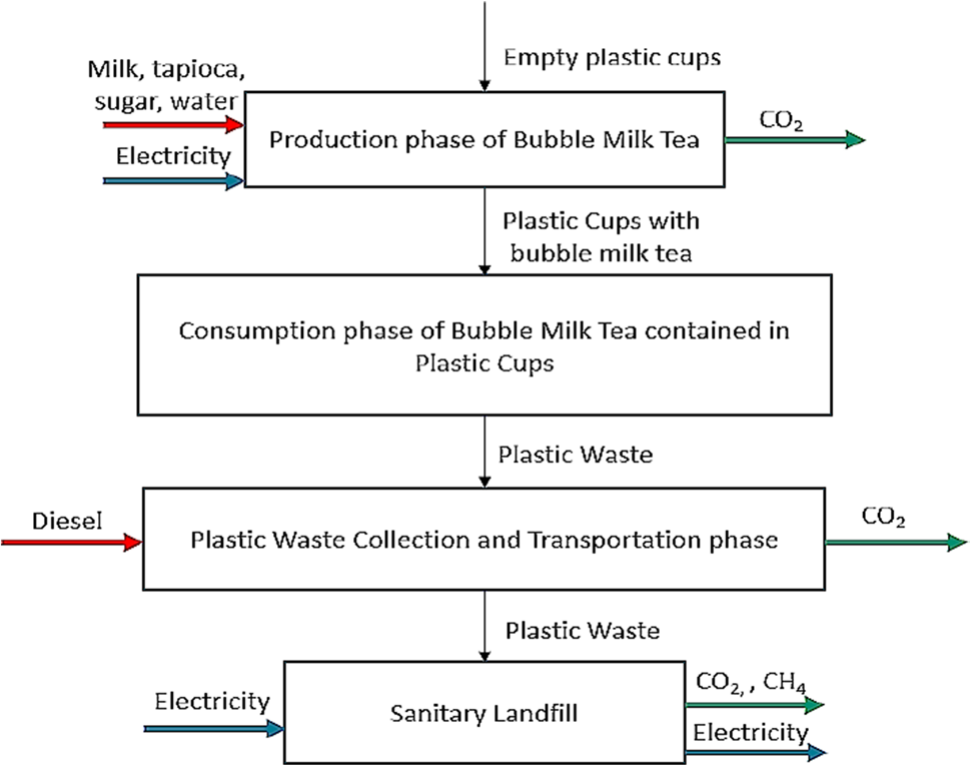
System boundary of existing plastic waste management for Scenario A

**Fig. 2:**
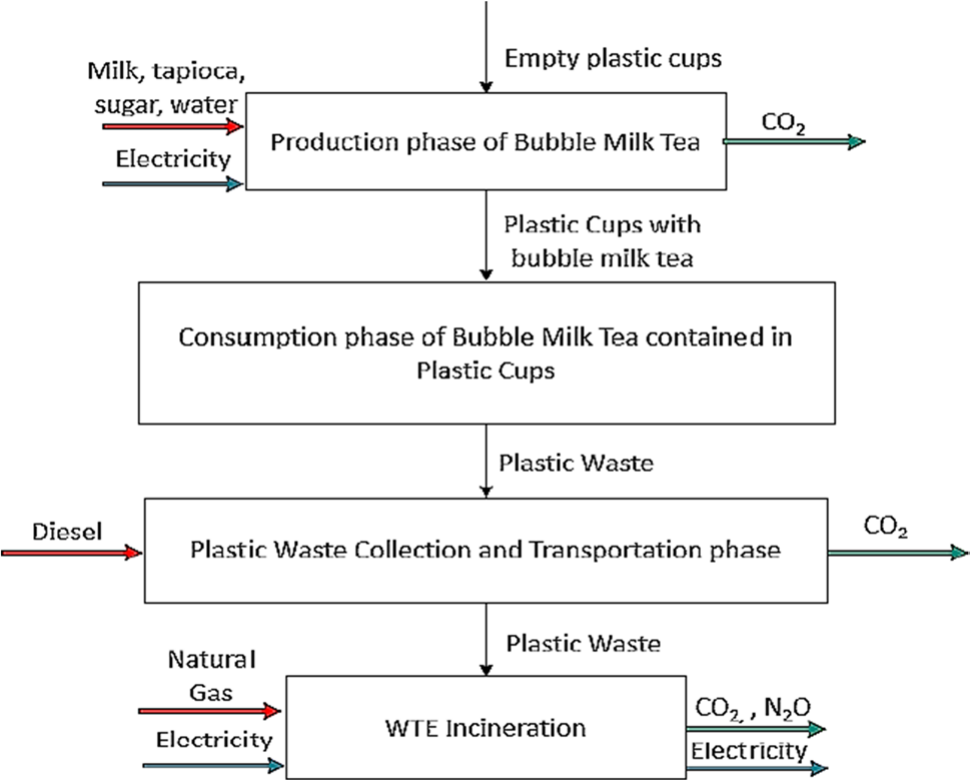
System boundary of plastic waste management for Scenario B

The goal of this LCA study is to achieve the aim of this study by quantifying the GHG emission of the plastic waste from bubble milk tea industry to the existing and alternative waste management scenarios (*Scenario A: Sanitary landfill* and *Scenario B: WTE incineration*) via gate-to-grave variant. These two waste management scenarios are to be compared for identification of better scenario with lowest net GHG emission. The functional unit is served to be the basis of comparison in the LCA system and the functional unit for this study is 1 tonne of plastic waste from Bubble Tea Streets. The system boundary of LCA includes four phases, which are production of bubble milk tea, consumption of bubble milk tea contained in plastic cups, plastic waste collection, and transportation and disposal of plastic waste. Figure 1 and Fig. 2 show the system boundaries of Scenarios A and B, respectively.

Please take note that plastic cup waste is termed as plastic waste in this study. There is no consumption of energy and emission during milk tea consumption by customer and material (plastic cup) is similar as input and output. Therefore, no inventory data analysis and impact assessment are conducted for this particular phase.

### Life Cycle Inventory

The life cycle inventory (LCI) is a collection of information related to four phases of LCA within the system boundary as shown in Fig. 2. The main primary data collected in this study are the plastic grade and types of electrical appliances used in the bubble tea shops. Secondary data such as resource and energy inputs as well as emission and energy outputs are obtained from journal articles and online websites.

### Data Related to Production of Bubble Milk Tea

Equation 1 calculates total electricity consumption of 26 bubble tea shops in the case study:

1 $$\Phi p=(\sum {W}_{i}\times {HR}_{\mathrm{Average}}\times {N}_{\mathrm{Shops}})$$

Equation 2 calculates CO_2_ emission from the total electricity consumption of these bubble tea shops:

2 $${E}_{{\mathrm{CO}}_{2},P}= {\Phi }_{P} \times {EF}_{\mathrm{mix}}$$

Inventory data as equation inputs related to production of bubble milk tea is tabulated in Table 3.

**Table 3:** Inventory data related to production of bubble milk tea

Input parameters	Unit	Data	Sources
Survey result			
${N}_{\mathrm{Shops}}$	**-**	26	
${HR}_{\mathrm{Average}}$	**-**	12	
Energy consumption of electrical appliances			
Sealer machine	W	400.00	USEPA 2020; Mike 2020)
Shaker machine		75.00	
Fructose dispenser		300.00	
Fridge		100.00	
Tube lights (× 6)		22.00 (× 6)	
Production emission			
${\mathrm{EF}}_{mix}$	kg CO_2_-eq/kWh	0.87	Logic 2019)

### Data Related to Waste Collection and Transportation

**Fig. 3:**
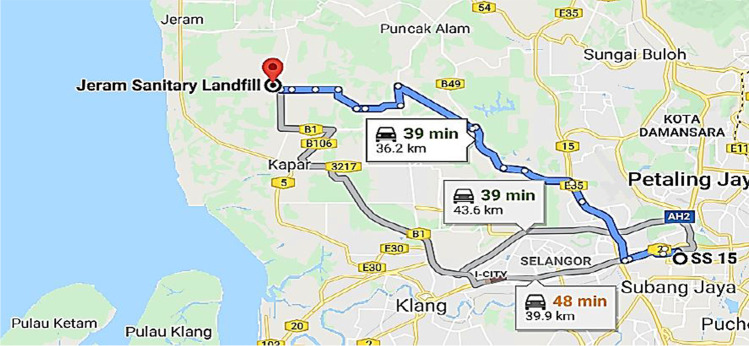
Map showing travel distance from Subang Jaya to Jeram Sanitary Landfill (Maps 2020)

Jeram Sanitary Landfill is responsible for MSW disposal from municipality of Subang Jaya, Malaysia (case study location) (Yong et al. 2019). Besides this, there is an ongoing project of constructing a WTE incinerator at the Jeram Sanitary Landfill site. Thus, the distance of transportation of 1 tonne of plastic waste from Subang Jaya to respective waste management technologies for both scenarios is the same. Considering back and forth by the transporter from Subang Jaya to the landfill site as shown in Fig. 3, the total two-way distance is 72.40 km and the diesel consumption is estimated as 10.14 l for distance of 72.40 km (Transport 2020).

Equation 3 calculates the CO_2_ emission from road transport (Eggleston et al. 2006).

3 $$E_{{\mathrm{CO}}_2,T}=\mathrm{Diesel}\;\mathrm{Consumption}\times{NCV}_{\mathrm{diesel}}\times{EF}_{\mathrm{diesel}}$$

Inventory data as equation inputs related to waste transportation is tabulated in Table 4.

**Table 4:** Inventory data related to plastic waste transportation

Scenario	Unit	Data	Sources
Diesel consumption	kg	8.62	Fan et al. 2019)
NCV_diesel_	MJ/kg	43.00	Ritchie and Roser 2017)
*EF*_diesel_	kg CO_2_-eq/TJ	74 100.00	

### Data Related to Sanitary Landfill

Plastic waste in Scenario A is sent to sanitary landfill in Jeram. Emission factor is used to determine the GHG emission from landfill. The emission factor used in this study is an estimated value for a period of 100 years which has taken both CH_4_ and CO_2_ into consideration (Eriksson and Finnveden 2009). Equation 4 is obtained from the combination of two sources to calculate the CO_2_ emission from landfill (Eggleston et al. 2006; Fan et al. 2019).

4 $${E}_{{\mathrm{CO}}_{2},L}=\left(\mathrm{PW }\times {EF}_{{\mathrm{CO}}_{2},L}\right)+{\Phi }_{L} \times {EF}_{\mathrm{mix}})$$

Volume of landfill gas emitted, ${{\varvec{Q}}}_{\mathbf{l}\mathbf{a}\mathbf{n}\mathbf{d}\mathbf{f}\mathbf{i}\mathbf{l}\mathbf{l}}$ has to be determined in order to determine the volume of CH_4_ in landfill gas. Volume composition of landfill gas is assumed to be containing 50 vol% CO_2_ and 50 vol% CH_4_ (USEPA 2020). Equation 5 is derived using density values of CH_4_ and CO_2_ and ${EF}_{{\mathrm{CO}}_{2},L}$.

5 $${EF}_{{\mathrm{CO}}_{2},L}\times \mathrm{PW}=\left(0.5\times {{\varvec{Q}}}_{\mathbf{l}\mathbf{a}\mathbf{n}\mathbf{d}\mathbf{f}\mathbf{i}\mathbf{l}\mathbf{l}}\times {\rho }_{{CO}_{2} }\times 1\right)+\left(0.5\times {{\varvec{Q}}}_{\mathbf{l}\mathbf{a}\mathbf{n}\mathbf{d}\mathbf{f}\mathbf{i}\mathbf{l}\mathbf{l}}\times {\rho }_{{CH}_{4}}\times 28\right)$$

Equation 6 calculates the amount of electricity generated from combustion of biogas captured by landfill gas recovery system (LGRS) (Alzate et al. 2019).

6 $$\mathrm{Electricity\;from }\;{\mathrm{CH}}_{4}\mathrm{\;combustion }\left(\mathrm{kWh}\right)={NCV}_{{\mathrm{CH}}_{4}}\times {Q}_{{\mathrm{CH}}_{4} }\times\upsigma \times {\eta }_{L}$$

Electricity generation from combustion of CH_4_ from LGRS of sanitary landfill is considered as renewable and cleaner energy. Thus, we can avoid the combustion of fossil fuels to create the same amount of energy, and the avoided emission from the combustion of fossil fuels is known as GHG saving. Equation 7 calculates GHG saving in terms of CO_2_ from electricity generation from LGRS as compared to similar amounts of electricity generation from the conventional power production in Malaysia (Eggleston et al. 2006; Fan et al. 2019).

7 $$E_{{\mathrm{CO}}_2,SL}=\left(\mathrm{Electricity}\;\mathrm{from}\;{\mathrm{CH}}_4\;\mathrm{combustion}\times{EF}_{\mathrm{mix}}\right)-(\mathrm{amount}\;of\;{\mathrm{CH}}_4\times{NCV}_{{\mathrm{CH}}_4}\times{EF}_{{\mathrm{CH}}_4})$$

Inventory data as equation inputs related to sanitary landfill is tabulated in Table 5.

**Table 5:** Inventory data related to sanitary landfill disposal of plastic waste

Input parameters	Unit	Data	Sources
Energy and material			
${\mathrm{Electricity}}_{L}$	kWh	11.11	Eriksson and Finnveden 2009)
Landfill emission			
${EF}_{{\mathrm{CO}}_{2},L}$	kg CO_2_-eq/t	271.00	Eriksson and Finnveden 2009)
Energy recovery			
σ	-	0.90	Alzate et al. 2019)
${\eta }_{L}$	-	0.38	Eriksson and Finnveden 2009)
Landfill gas property			
${\rho }_{{\mathrm{CH}}_{4}}$	kg/m^3^	0.66	Ritchie and Roser 2017)
${\rho }_{{\mathrm{CO}}_{2}}$	kg/m^3^	1.98	
${NCV}_{{\mathrm{CH}}_{4}}$	kWh/m^3^	9.94	Banister and Sullivan 2011)
	MJ/kg	50.00	
GHG saving			
${EF}_{{\mathrm{CH}}_{4}}$	kg CO_2_-eq/TJ	54,600.00	Ritchie and Roser 2017)
${EF}_{\mathrm{mix}}$	kg CO_2_-eq/kWh	0.87	Logic 2019)

### Data Related to WTE Incineration

Scenario B has the plastic waste sent to the WTE incinerator to reduce the dependence on landfills. Equation 8 is obtained from combination of two sources to calculate CO_2_ emission from incineration (Eggleston et al. 2006; Fan et al. 2019).

8 $${E}_{{\mathrm{CO}}_{2},I}=\left(\mathrm{PW}\times \mathrm{Dm}\times \mathrm{CF}\times \mathrm{FCF}\times \mathrm{OF}\times \frac{44}{12}\right)+\left({\Phi }_{I}\times {EF}_{\mathrm{mix}}\right)+({\mathrm{Fuel}}_{I}\times {EF}_{\mathrm{gas}})$$

Equation 9 calculates N_2_O emission from incineration (Eggleston et al. 2006).

9 $${E}_{{N}_{2}O,I}=\mathrm{PW}\times {EF}_{{N}_{2}O,I}$$

The energy recovery from incineration in terms of electricity can be calculated using Eq. 10 (Alzate et al. 2019). Based on survey results, all 26 bubble tea vendors in Subang Jaya utilise polypropylene (PP) grade 5 plastic as a cup material. Hence, net calorific value of plastic in Eq. 10 is the net calorific value of PP plastic.

10 $$\mathrm{Electricity}\;\mathrm{from}\;\mathrm{Incineration}\;\left(\mathrm{kWh}\right)=\mathrm{PW}\times{NCV}_{PP}\times\eta_I$$

Equation 11 calculates GHG saving in terms of CO_2_-eq from electricity generation from the WTE incinerator as compared to the similar amount of electricity generated from conventional power production (Fan et al. 2019).

11 $$E_{{\mathrm{CO}}_2,SI}=\left(\mathrm{Electricity}\;\mathrm{from}\;\mathrm{Incineration}\times{EF}_{\mathrm{mix}}\right)$$

Inventory data as equation inputs related to WTE incineration is tabulated in Table 6.

**Table 6:** Inventory data related to WTE incineration of plastic waste

Input parameters	Unit	Data	Sources
Energy and material			
${\mathrm{Electricity}}_{I}$	kWh	70	Toolbox 2003)
${\mathrm{Fuel}}_{I}$	MJ	9.86	
Incineration emission			
${EF}_{\mathrm{gas}}$	kg CO_2_-eq/TJ	56,100.00	Ritchie and Roser 2017)
${EF}_{{\mathrm{N}}_{2}\mathrm{O},I}$	g N_2_O/t	47	
Plastic property			
Dm	-	0.93	Logic 2019)
CF	-	0.86	
FCF	-	0.69	
OF	-	1.00	
Energy recovery			
${NCV}_{PP}$	kWh/kg	11.39	Khoo 2019)
${\eta }_{I}$	-	0.30	Caneghem et al. 2019)
GHG saving			
${EF}_{\mathrm{mix}}$	kg CO_2_-eq/kWh	0.87	Logic 2019)

### Life Cycle Impact Assessment

Global warming potential (GWP) is the only indicator used in this case study as the GHGs are the main emission from all phases of LCA. GWP represents the amount of carbon dioxide (CO_2_) and other greenhouse gases (GHGs) emitted over a full life cycle of a process or a product. GHGs such as CH_4_ and N_2_O are converted to CO_2_-eq by multiplying with their respective GWP as shown in Table 7.

**Table 7:** Equivalency factor for global warming (Tsiamis and Castaldi 2016)

GHG	100-year GWP (CO_2_-eq)
CO_2_	1
CH_4_	28
N_2_O	265

## Results and Discussion

### Scenario A: Baseline Scenario—Sanitary Landfill

**Fig. 4:**
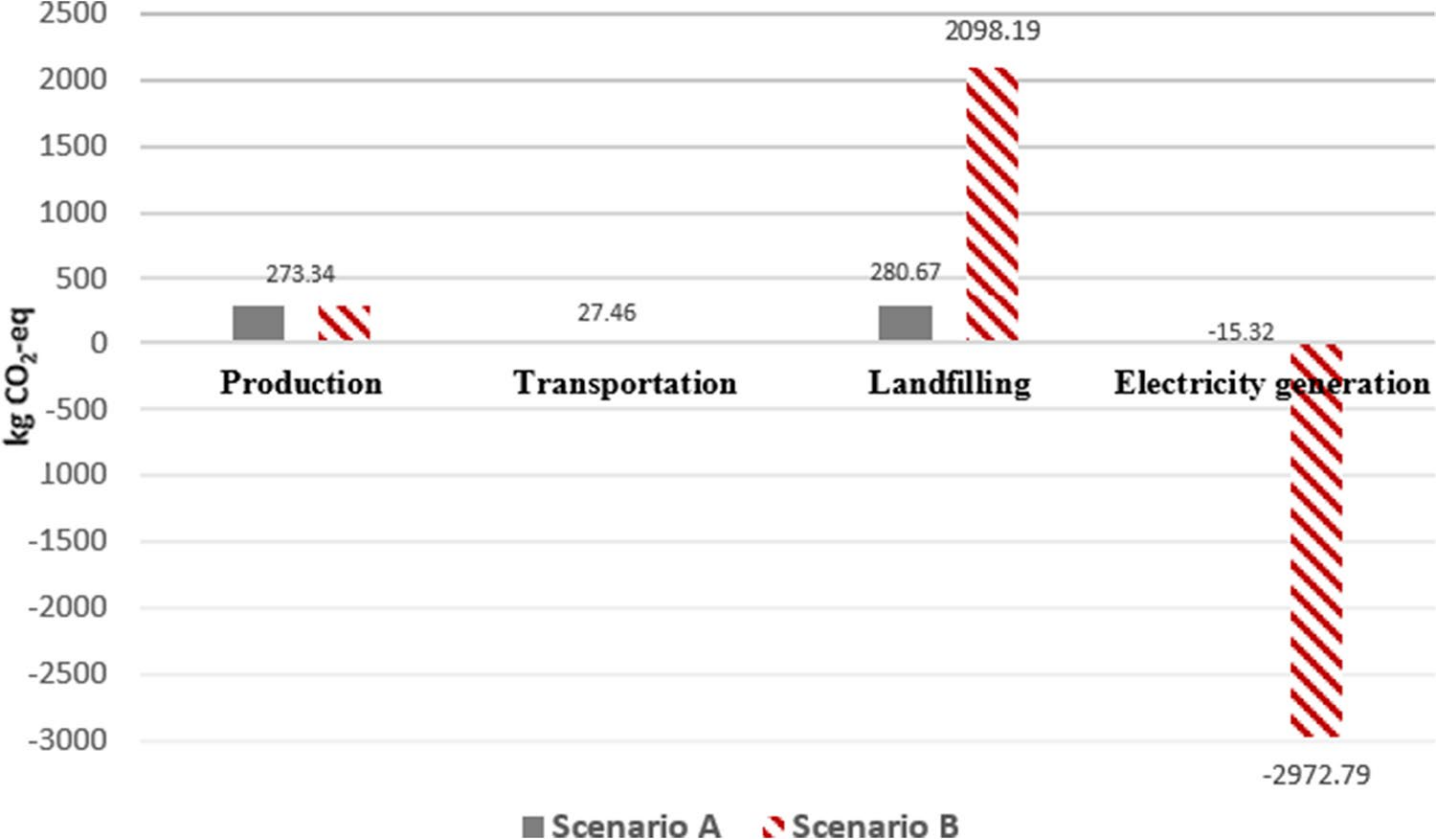
GHG emission of the activities of 1 tonne of plastic waste for Scenarios A and B

Based on GHG results tabulated in Table 8 and plotted in Fig. 4, landfilling had the highest GHG emission as compared to other phases within the system boundary of this LCA study due to potent CH_4_ emission. Many sanitary landfills in Malaysia have HDPE geomembrane as bottom liners to achieve landfill gas collection efficiency up to 90% (Banister and Sullivan 2011). Despite LGRS has a high efficiency to capture CH_4_ combustion purpose, the uncaptured CH_4_ is still a main concern as emission of 1 kg of CH_4_ is equivalent to emission of 28 kg CO_2_ into the environment. The net GWP/GHG emission for sanitary landfill scenario was 566.15 kg CO_2_-eq per tonne of plastic waste after taking into consideration the GHG saving of 15.32 kg CO_2_-eq achieved from the electricity generation from combustion of captured CH_4_.

**Table 8:** Net GHG emission for sanitary landfill disposal of 1 tonne of plastic waste (Scenario A)

LCA phases	Unit	Values
(A) Production of bubble milk tea		
Electricity consumption for daily operation of bubble tea shops in Subang Jaya	kWh	314.18
Total GHG for production of bubble milk tea	kg CO_2_-eq	**273.34**
(B) Transportation		
Diesel consumption for transportation of plastic waste from Bubble Tea Street in Subang Jaya to Jeram Sanitary Landfill for two way distance of 72.4 km	L	10.14
Total GHG for transportation	kg CO_2_-eq	27.46
(C) Landfilling		
Electricity consumption for compacting the waste	kWh	11.11
CO_2_ emission from electricity consumption	kg CO_2_-eq	9.67
CO_2_ emission from landfill	kg CO_2_-eq	26.23
CH_4_ emission (convert to GWP unit)	kg CO_2_-eq	244.77
Total GHG for landfilling	kg CO_2_-eq	**280.67**
Gross GHG emission	kg CO_2_-eq	**581.47**
(D) Electricity generation		
Electricity generated	kWh	45.03
CO_2_ emission from CH_4_ combustion	kg CO_2_-eq	23.86
CO_2_ emission based on electricity generation mix	kg CO_2_-eq	− 39.18
Total GHG saving	kg CO_2_-eq	− **15.32**
Net GHG emission (A + B + C + D)	kg CO_2_-eq	**566.15**

A new study has found out that CH_4_ emission from plastic waste has continued at night once the plastic waste is exposed to ambient solar radiation and the exposed aged plastic will release more CH_4_ at night (Shen et al. 2020). CH_4_ emission from this new study is neither accounted in the journal articles and LCA software nor accounted by the equations listed by IPCC; therefore, the actual GHG emission from landfilling was expected to be higher than the calculated value of 280.67 kg CO_2_-eq per tonne of plastic waste being disposed into sanitary landfills as shown in Table 8. Electricity consumption from production of bubble milk tea was ranked second in terms of GHG emission which has contributed to 273.34 kg CO_2_-eq followed by small amount of GHGs released from mobile combustion of diesel for transportation and stationary combustion of CH_4_ for electricity generation. These four activities have combined to give the gross GWP/GHG emission of 581.47 kg CO_2_-eq.

Up to date, only 6% of the landfills in Malaysia are sanitary and as a result, landfilling is still the primary CH_4_ emission source in Malaysia. Therefore, the first step for local authorities to implement sustainable plastic waste management plan is to phase out non-sanitary landfills or upgrade these landfills into sanitary ones. With LGRS featured into these upgraded landfills, GHG emission can be reduced by 15.32 kg CO_2_-eq for every tonne of plastic waste that is being disposed into landfill.

### Scenario B: WTE Incineration

Based on Table 9 and Fig. 4, the GHG emissions from production of bubble milk tea, transportation, and incineration of plastic waste have combined to generate a gross GWP of 2398.99 kg CO_2_-eq. The GHG emissions from the production phase of bubble milk tea and transportation phase of plastic waste in Scenario B were similar to the values obtained from Scenario A as expected as the input data for LCA study of these two phases are similar for both scenarios.

**Table 9:** Net GHG emission of WTE incineration of 1 tonne of plastic waste for Scenario B

LCA phases	Unit	Values
(A) Production of bubble milk tea		
Electricity consumption for daily operation of bubble tea shops in Subang Jaya	kWh	314.18
Total GHG for production of bubble milk tea	kg CO_2_-eq	**273.34**
(B) Transportation		
Diesel consumption for transportation of plastic waste from Bubble Tea Street in Subang Jaya to Jeram Sanitary Landfill for two way distance of 66.4 km	L	10.14
Total GHG for transportation	kg CO_2_-eq	**27.46**
(C) Incineration		
Natural gas consumption as auxiliary fuel for process startup	MJ	9.86
CO_2_ emission from fuel consumption	kg CO_2_-eq	0.55
Electricity consumption for process startup	kWh	70
CO_2_ emission from electricity consumption	kg CO_2_-eq	60.90
CO_2_ emission from incineration	kg CO_2_-eq	2023.49
N_2_O emission (convert to GWP unit)	kg CO_2_-eq	13.25
Total GHG for incineration	kg CO_2_-eq	**2098.19**
Gross GHG emission	kg CO_2_-eq	**2398.99**
(D) Electricity generation		
Electricity generated	kWh	3417.00
CO_2_ emission based on electricity generation mix	kg CO_2_-eq	− 2972.79
Total GHG saving	kg CO_2_-eq	− **2972.79**
Net GHG emission (A + B + C + D)	kg CO_2_-eq	− **573.80**

Incineration process had the highest GHG emission as compared to other phases and the emission was greater than the main GHG contributor in Scenario A—landfilling by 1817.52 kg CO_2_-eq. The high GHG emission is due to higher net calorific value of the plastic. The PP plastic has a net calorific value of 41 MJ/kg which is about four times greater than the average calorific value of MSW in Malaysia of 10.88 MJ/kg (Yong et al. 2019; Tsiamis and Castaldi 2016). The higher the calorific value of waste, the greater the amount of heat energy that can be extracted from the waste, and this leads to higher GHG emission. However, the actual GHG emission from incineration will be much lower as the equations provided by IPCC guidelines did not take into account reduction in carbon emission by advanced air pollution control system such as scrubber and activated carbon filter that are featured in the modern WTE incinerators. Nevertheless, the net GWP of WTE incineration was − 573.80 kg CO_2_-eq and the huge reduction in GWP was contributed by huge GHG saving being achieved from electricity generation by the WTE incinerator which is much cleaner than conventional power production practice of Malaysia. Astonishing amounts of 3.42 MWh of electricity were generated due to high net calorific value of plastic waste and this has led to a GHG saving of 2972.79 kg CO_2_-eq. Higher amounts of electricity can be generated and greater GHG saving can be obtained if the electrical conversion efficiency of the WTE incinerator was higher than 30%.

### Overall Result of Plastic Waste Management Scenarios

**Fig. 5:**
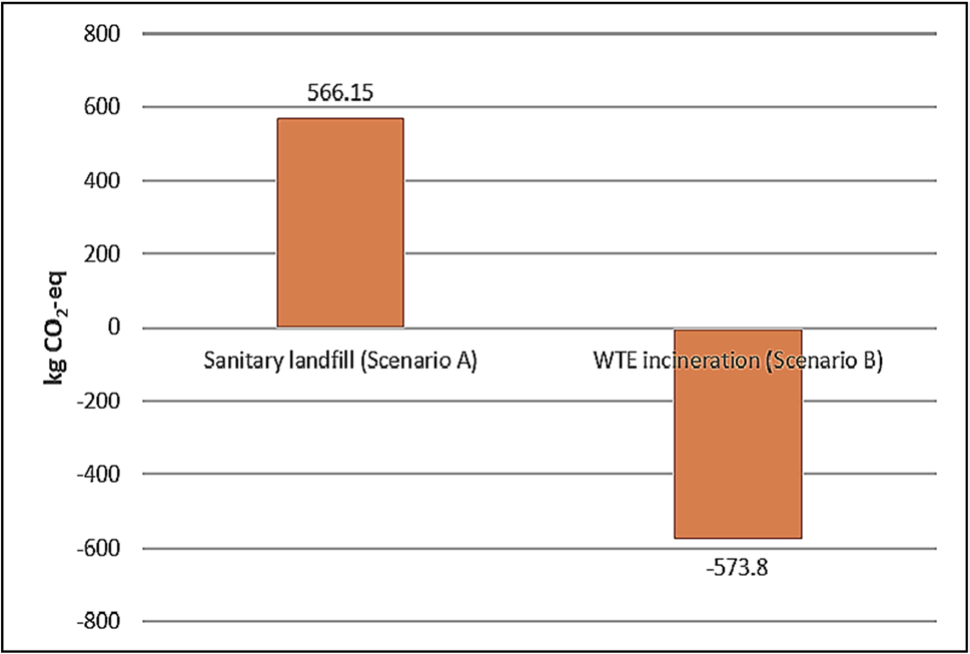
Comparative study of net GWP for different plastic waste management scenarios

Based on net GHG emission/GWP for two plastic waste management scenarios as shown in Fig. 5, the overall comparison of both scenarios indicated that Scenario B (WTE incineration) was the better scenario in terms of environmental sustainability where it had a negative net GWP and it was able to achieve GHG reduction of 1139.95 kg CO_2_-eq/t of plastic waste being sent to WTE incineration instead of the Scenario A (sanitary landfill). Besides this, WTE incineration was able to generate 3.42 MWh of electricity per tonne of plastic waste being incinerated as compared to sanitary landfill where only 45.03 kWh of electricity was generated for every tonne of plastic waste being landfilled. Moreover, plastic waste disposal phase was the primary hotspot for GWP in both scenarios as the waste management technologies and electricity generation are both under plastic waste disposal phase which had significant impacts on net GHG emission.

It is better to keep GHG emission of N_2_O and CH_4_ as low as possible because these gases have higher GWP. CH_4_ is the main GHG from landfilling whereas N_2_O is the main GHG from incineration. Although the GWP of N_2_O is 9.46 times greater than CH_4_, mass fraction of N_2_O only accounts for 0.78% of the hot flue gas exiting the WTE incinerator as opposed to sanitary landfill where CH_4_ accounts for 25% of total mass fraction of landfill gas. When comparing both GHG emission per tonne of plastic waste in terms of kg CO_2_-eq, CH_4_ emission from sanitary landfill and N_2_O emission from the WTE incinerator were 244.77 kg CO_2_-eq and 13.25 kg CO_2_-eq, respectively. As a result, WTE incineration is a more environmentally friendly choice if the study was to focus on the reduction of GWP attributed by these two potent GHGs.

In the incineration process, combustion of plastic waste under high temperature generates huge amount of CO_2_ which contributes significantly to global warming and rapid climate change. However, the high GWP is compensated by GHG saving via electricity generation from incineration where the emission is lower than conventional power production. While the electricity conversion efficiency of both sanitary landfill and WTE incinerator is within the range of 30 to 40%, the energy recovery from incineration was much greater than landfill due to higher electricity generation. The recovery difference is mainly due to the amount of fuels being combusted in both energy recovery systems. Energy recovery of WTE incinerator was greater as compared to sanitary landfill as the entire 1 tonne of plastic waste was consumed and converted to heat energy, unlike LGRS of sanitary landfill where only the CH_4_ composition of captured landfill gas (8.74 kg) produced from landfill disposal of 1 tonne of plastic waste was consumed and converted to heat energy with remaining CO_2_ and uncaptured CH_4_ were released to the environment. Moreover, characteristic of CH_4_ is almost similar to natural gas in which there is only slight GHG saving when comparing emission from electricity generated from combustion of CH_4_ with conventional power production of Malaysia.

Based on the LCA result on GHG emission, WTE incineration can be an effective alternative to improve plastic waste management as it can divert more plastic waste from the sanitary landfill and offer GHG saving through electricity generation from plastic waste. In addition, the average net calorific value of all 7 grades of petroleum-based plastic is 35.7 MJ/kg or 9.92 kWh/kg and GHG saving of 2589.12 kg CO_2_-eq can be achieved with the net GHG being remained as a negative figure with the amount of − 190.13 kg CO_2_-eq if the average net calorific value of all plastic grades was used for the environmental impact assessment of Scenario B instead of using net calorific value of PP plastic alone (Tsiamis and Castaldi 2016).

In conclusion, WTE incineration treatment was proven as a carbon-negative technology and can be used as the basis of environmental sustainability framework development for plastic waste management to tackle single-use plastic waste from Bubble Tea Streets of Subang Jaya in order to create a cleaner environment and to prevent the increase in global temperature caused by single-use plastics. The main contributor that has led to a negative net GHG emission in the overall environmental performance of WTE incineration was the huge GHG saving achieved from the renewable electrical energy generated from incineration. This is because Malaysia is highly depending on fossil fuels for electricity generation and the GHG emission to create useful energy from conventional power production is much greater as compared to WTE incineration. Hence, the power production practice of Malaysia has a significant impact on net GHG emission of WTE incineration. The environmental feasibility of implementing WTE technology might be altered in the future if the renewable energy sources have higher share in the power production practice.

### Environmental Sustainability Framework for Plastic Waste Management in Malaysia

Growth of food and beverage industries such as the bubble tea industry has accelerated the generation of single-use plastic waste. These low-quality petroleum-based plastics take longer time to degrade as compared to other types of solid waste and thus, taking up more spaces in landfills in the long run. As a result, the government is seeking for alternative WTE incineration to replace landfills for plastic waste disposal but the environmental performance of this alternative is still unknown. However, it was proven from an LCA study that a negative net GWP of − 573.80 kg CO_2_-eq was achieved for every tonne of plastic waste being sent to the WTE incinerator, a further confirmation that WTE incineration is more environmental friendly than sanitary landfill.

Materialising a plastic waste management framework in a sustainable way is a challenging and difficult task which cannot simply be done by the government alone. Besides reducing global warming impact and the dependence on landfill with implementation of WTE incineration, the responsibility to ensure a successful and effective development plastic waste management framework is shared among the government, bubble tea vendors, waste contractors, and consumers. This framework assumes bubble tea business is carried out in normal course of business without being affected by the coronavirus pandemic.

The government plays an important role in ensuring continuity of a proposed environmentally sustainable plastic waste management framework. Besides enforcing stringent regulations on plastic littering, collection and sorting of plastic waste by vendors, as well as controlling air emission from incinerators, the government can allocate more plastic waste bins around Bubble Tea Streets to provide convenience to customers for disposing the plastic cups after consumption of bubble milk tea. Besides, the government can also encourage these bubble tea vendors to seek alternatives to phase out single-use plastic cups. Alternatively, the government can enforce a total ban on single-use plastics which is proven to be very effective to indirectly forcing the vendors to utilise environmental friendlier cup material as soon as possible (Ali et al. 2021). Next, the government can appoint reputable waste collector company to collect waste from collection bins to ensure Subang Jaya is always free of plastic waste before transporting it to the waste management company.

Establishing a WTE incinerator requires high capital cost and the government can aid in capital in the form of waste management subsidy or grant. The government can then enter into a contract agreement with waste management company, which is the owner of WTE incinerator in this case, to agree on duration of its operation and ensure standard fees are charged for waste disposal. Municipal plastic waste is considered as renewable biomass source and the government pays money directly to the waste management company in exchange for electricity that is being sold to Tenaga National Berhad (TNB) in accordance to the Feed-In Tariff (FIT) rates provided by the Sustainable Energy Development Authority (SEDA) Malaysia (SEDA 2020). Part of this renewable energy can be supplied to the bubble tea vendors in Subang Jaya to run their daily business activities which require an average of 314.18 kWh of electricity. Thus, a conclusion can be made in which more electricity can be generated and greater GHG saving can be achieved if more single-use plastic wastes are being incinerated instead of landfilling. According to waste management hierarchy, reduce and reuse are better waste management options as compared to waste treatment and disposal. Therefore, plastic waste should be reduced instead of sending the waste to incinerator for electricity generation without taking any initiative to reduce the GHG emission from incineration. Bubble tea vendors can do their parts in reducing plastic waste and to spread plastic pollution awareness among the public by utilising paper or biodegradable plastic cups to phase out single-use plastics. Moreover, bubble tea vendors can encourage customers to bring their own tumblers by giving them incentives such as discount or drink rewards thus cutting down plastic waste pollution. Furthermore, bubble tea shops can also introduce reusable tumblers.

The role of a waste management company is to keep the discharged CO_2_ as low as possible in order to comply with the stringent regulation set by the Department of Environment (DOE). With the financial support from the government, further upgrades can be done on the existing advanced air pollution control system which are featured in the WTE incinerator to further reduce the GHG emission coming out from the chimney.

**Fig. 6:**
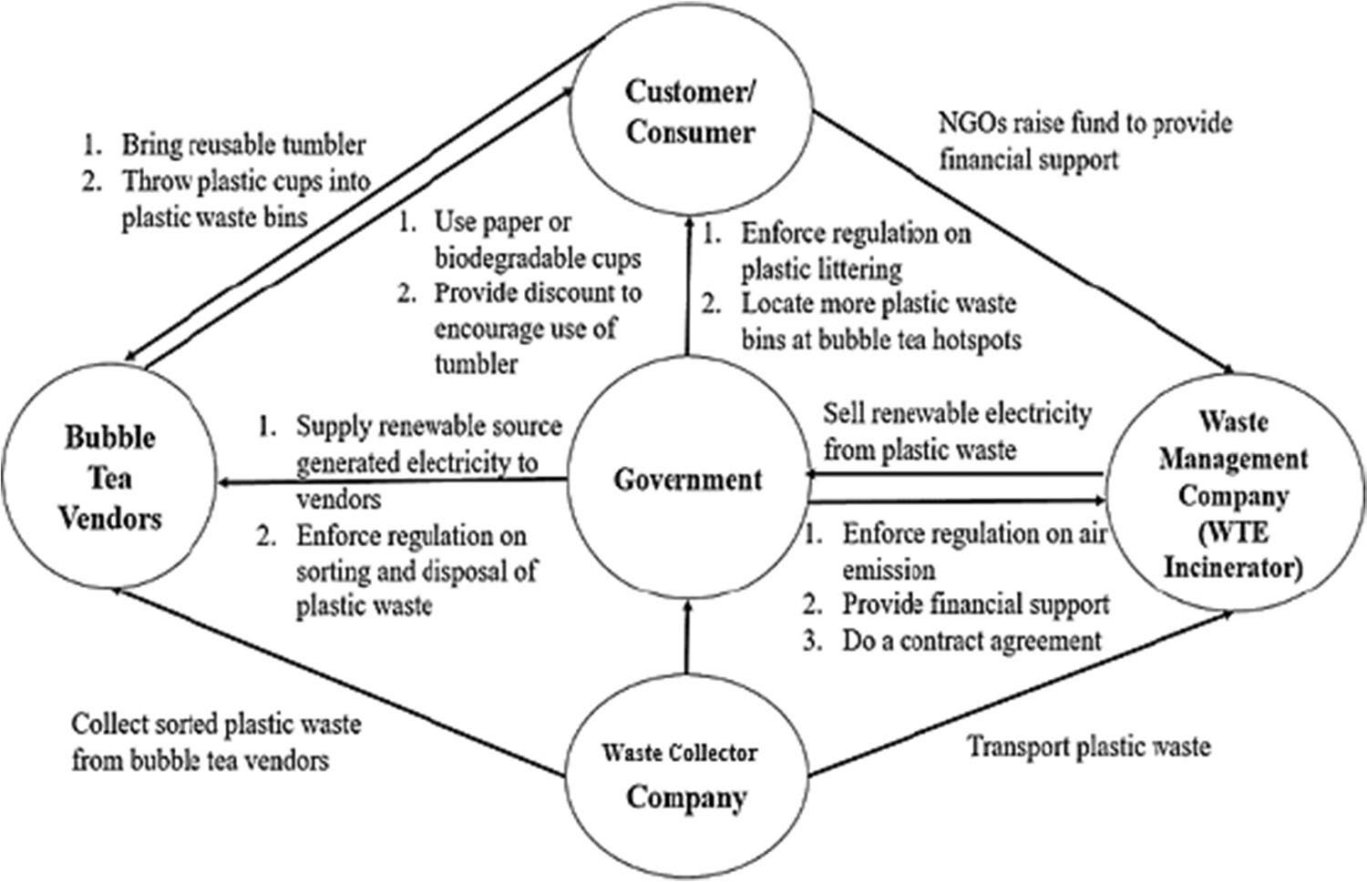
Proposed environmental sustainability framework for plastic waste management in Malaysia (F&B industry)

The proposed environmental sustainability framework for plastic waste management in Subang Jaya can be further visualised in Fig. 6. The framework highlights on the role of each party to manage and reduce the GHG emission associated with plastic waste. The framework also suggests alternative ways to reduce the use of plastics.

## Conclusion

Based on the study, the GHG emissions in terms of CO_2_-eq of sanitary landfill (Scenario A) and WTE incineration (Scenario B) were 566.15 kg CO_2_-eq and − 573.80 kg CO_2_-eq, respectively. Hence, WTE incineration was proven to be more environmentally friendly as compared to sanitary landfill due to the huge GHG saving achieved from cleaner electricity generation via incineration as compared to the current power production practice of Malaysia. The result suggested that GHG saving could be further increased if energy conversion efficiency of WTE incineration can be increased to be more than 30% for greater electricity generation.

The proposed environmental sustainability framework for plastic waste management was to showcase a general idea for the Malaysian government to deal with increasing solid waste issue, especially on plastic waste caused by heavy usage of single-use plastics from the bubble tea industry. The outcome of this study enables to serve as a benchmark in plastic waste disposal management in Malaysia. If this framework was deemed to be successful upon its implementation, it can be considered by the government to implement on other plastic waste generation hotspots other than bubble tea industry or as a reference to other nations to deal with plastic waste issue generated from the F&B industry. However, the transition from sanitary landfills to WTE incineration should not be hurried. Comprehensive information about their applicability could be included for consideration in order to provide a more holistic assessment of sustainability feasibility. Although GWP is the main environmental impact indicator when dealing with plastic waste, other minor environmental impact indicators should be taken into consideration to assess the overall environmental performance of existing sanitary landfill and alternative WTE incinerator. Furthermore, a more comprehensive study such as economic and social aspects of WTE incineration and sanitary landfill should be taken into consideration to help policy makers to implement a better Twelfth Solid Waste Management Plan (2021–2025) which benefits all parties that are dealing with plastic waste issue.

## Data Availability

Datasets used and/or analysed during the study are available from the author on reasonable request.

## List of symbols

*Φ*_*p*_: Total electricity consumption for production of bubble milk tea (kWh)
${W}_{i}$: Power rating of each electrical appliance (kW)
${HR}_{\mathrm{Average}}$: Average operating hours (h)
${N}_{\mathrm{Shops}}$: Number of bubble tea shops
${E}_{{\mathrm{CO}}_{2},P}$: CO_2_ emission from electricity consumption (kg CO_2_-eq)
${\mathrm{EF}}_{\mathrm{mix}}$: Emission factor of CO_2_ based on electricity generation mix in Malaysia (kg CO_2_-eq/kWh)
${E}_{{\mathrm{CO}}_{2},T}$: CO_2_emission from transportation (kg CO_2_-eq)
${NCV}_{\mathrm{diesel}}$: Net calorific value of diesel (MJ/kg)
*EF*_diesel_: Emission factor of CO_2_ from diesel combustion (kg CO_2_-eq/TJ)
${E}_{{\mathrm{CO}}_{2},L}$: CO_2_ emission from landfill (kg CO_2_-eq)
PW: Plastic waste (t)
${EF}_{{CO}_{2},L}$: Emission factor of CO_2_ from landfill (kg CO_2_-eq/t)
*Φ*_*L*_: Electricity consumption for compacting the waste in landfill (kWh)
${\rho }_{{\mathrm{CO}}_{2}}$: Density of CO_2_ (kg/m^3^)
${\rho }_{{\mathrm{CH}}_{4}}$: Density of CH_4_ (kg/m^3^)
${NCV}_{{\mathrm{CH}}_{4}}$: Net calorific value of CH_4_ (kWh/m^3^)
*Q*_CH4_: Volume of CH_4_ in landfill gas (m^3^)
*σ*: Capturing efficiency of landfill gas recovery system (LGRS)
${\eta }_{L}$: Electrical energy production efficiency of LGRS
${E}_{{\mathrm{CO}}_{2},SL}$: GHG saving from electricity generation from LGRS (kg CO_2_-eq)
${EF}_{{\mathrm{CH}}_{4}}$: Emission factor of CO_2_ from CH_4_ combustion (kg CO_2_-eq/TJ)
${E}_{{\mathrm{CO}}_{2},I}$: Emission of CO_2_ from incineration (kg CO_2_-eq)
*D*_m_: Dry matter content of plastic waste
CF: Fraction of carbon in dry matter
FCF: Fraction of fossil carbon
OF: Oxidation factor (fraction)
44/12: Conversion factor from C to CO_2_
*Φ*_*I*_: Electricity consumption for startup of incinerator
${\mathrm{Fuel}}_{I}$: Natural gas consumption for startup of incinerator (MJ)
${EF}_{\mathrm{gas}}$: Emission factor of CO_2_ from natural gas combustion (kg CO_2_-eq/TJ)
${E}_{{\mathrm{N}}_{2}\mathrm{O},I}$: Emission of N_2_O from incineration (kg N_2_O)
${EF}_{{\mathrm{N}}_{2}\mathrm{O},I}$: Emission factor of N_2_O from incineration (kg N_2_O/t)
${NCV}$: Net calorific value (kWh/kg)
${\eta }_{I}$: Electrical energy production efficiency of incineration
${E}_{{\mathrm{CO}}_{2},SI}$: GHG saving from electricity generation from WTE incinerator (kg CO_2_-eq

## References

[CR1] Abdullah WSW, Osman M, Kadir MZAA, Verayiah R (2019). The potential and status of renewable energy development in Malaysia. Energies.

[CR2] Akenji L, Bengtsson M, Hotta Y, Kato M, Hengesbaugh M (2020). Policy responses to plastic pollution in Asia: summary of a regional gap analysis. Plastic Waste and Recycling.

[CR3] Ali S, Ahmed W, Solangi YA, Zarei N, Chaudhry IS (2021). Strategic analysis of single-use plastic ban policy for environmental sustainability: the case of Pakistan. Clean Technol Environ Policy.

[CR4] Alias FS, Manaf A, Latifah A, Mariani & Abdullah, Ho S,  (2018). Solid waste minimization in Malaysia. Pertanika Journal of Scholarly Research Reviews.

[CR5] Alzate S, Cuestas BR, Duque AJ (2019). Municipal solid waste as a source of electric power generation in Colombia: a techno-economic evaluation under different scenarios. Resources.

[CR6] Arvanitoyannis IS (2008) ISO 14040: life cycle assessment (LCA)–principles and guidelines. Waste management for the food industries 97–132.

[CR7] Aziz A (2020) Another due date for country’s 1st WTE plant. The Malaysian Reserve. https://themalaysianreserve.com/2020/09/23/another-due-date-for-countrys-1st-wte-plant/#:~:text=The%20due%20date%20was%20then,the%20operation%20for%20three%20months. Accessed 22 December 2020

[CR8] Banister AVK, Sullivan PS (2011). LFG collection efficiency: debunking the rhetoric. MSW Management.

[CR9] CCET guideline series on intermediate municipal solid waste treatment technologies: waste-to-energy incineration (2020) Institute for Global Environmental Strategies, Tokyo, Japan

[CR10] Chang L, Tan J (2021). An integrated sustainability assessment of drinking straws. J. Environ. Chem. Eng.

[CR11] Eggleston S, Buendia L, Miwa K, Ngara T & Tanabe K (2006) 2006 IPCC guidelines for national greenhouse gas inventories. Institute for Global Environmental Strategies, Hayama, Japan

[CR12] Eriksson O, Finnveden G (2009). Plastic waste as a fuel-CO 2-neutral or not?. Energy Environ Sci.

[CR13] Fan YV, Klemeš JJ, Lee CT, Perry S (2019). GHG emissions of incineration and anaerobic digestion: electricity mix. Chem Eng Trans.

[CR14] Geyer R, Jambeck JR, Law KL (2017). Production, use, and fate of all plastics ever made. Sci Adv.

[CR15] Kaza S, Yao L, Bhada-Tata P & Van Woerden F (2018) What a waste 2.0: a global snapshot of solid waste management to 2050. The World Bank, Washington, DC

[CR16] Khoo HH (2019). LCA of plastic waste recovery into recycled materials, energy and fuels in Singapore. Resour Conserv Recycl.

[CR17] KPKT (2015) Solid waste management lab 2015: final lab report. Ministry of Housing and Local Government (KPKT), Putrajaya, Malaysia

[CR18] Lew R (2020) Moving grate incineration: preferred WTE technology. Bioenergy Consult. https://www.bioenergyconsult.com/moving-grate-incineration/. Accessed 22 December 2020

[CR19] Draft Logic (2019) List of the power consumption of typical household appliances. https://www.daftlogic.com/information-appliance-power-consumption.htm. Accessed 10 December 2020

[CR20] Google Maps (2020) Travel distance from SS15, Subang Jaya to Jeram Sanitary Landfill

[CR21] Mike R (2020) Bubble tea store design layout. Bubble Teaology. https://www.bubbleteaology.com/bubble-tea-store-design-layout/. Accessed 10 December 2020

[CR22] Moy CH, Tan LS, Shoparwe NF, Shariff AM, Tan J (2021). Comparative study of a life cycle assessment for bio-plastic straws and paper straws: Malaysia’s perspective. Processes.

[CR23] Ncube LK, Ude AU, Ogunmuyiwa EN, Zulkifli R, Beas IN (2021). An overview of plastic waste generation and management in food packaging industries. Recycling.

[CR24] Perrot JF, Subiantoro A (2018). Municipal waste management strategy review and waste-to-energy potentials in New Zealand. Sustainability.

[CR25] Ritchie H & Roser M (2017) CO_2_ and greenhouse gas emissions. Our World in Data. https://ourworldindata.org/co2-and-other-greenhouse-gas-emissions?fbclid=IwAR1-222h-a-w2N6erxdE8kSEBVGCY0XZnxaFhhG8R4KjvPaEwlrXA0sMkxk. Accessed 15 December 2020

[CR26] Ritchie H & Roser M (2018) Plastic pollution. Our World in Data. https://ourworldindata.org/plastic-pollution. Accessed 15 December 2020

[CR27] SEDA (2020). Feed-In Tariff (FIT). http://www.seda.gov.my/reportal/fit/. Accessed 27 December 2020

[CR28] Shen M, Huang W, Chen M, Song B, Zeng G, Zhang Y (2020). (Micro) plastic crisis: un-ignorable contribution to global greenhouse gas emissions and climate change. J Clean Prod.

[CR29] Tan ST, Ho WS, Hashim H, Lee CT, Taib MR, Ho CS (2015). Energy, economic and environmental (3E) analysis of waste-to-energy (WTE) strategies for municipal solid waste (MSW) management in Malaysia. Energy Convers Manage.

[CR30] Tan J, Tan RR, Aviso KB, Promentilla MAB, Nik Sulaiman NM (2017). Study of microalgae cultivation systems based on integrated analytic hierarchy process–life cycle optimization. Clean Technol Environ Policy.

[CR31] Tan MZ (2019) Malaysia has 74 bubble tea brands, can you tell them all apart? Malay Mail. https://www.malaymail.com/news/life/2019/07/24/malaysia-has-74-bubble-tea-brands-can-you-tell-them-all-apart/1774311. Accessed 22 December 2020

[CR32] Engineering Toolbox (2003) Fuels - higher and lower calorific values. https://www.engineeringtoolbox.com/fuels-higher-calorific-values-d_169.html. Accessed 12 December 2020

[CR33] Transport (2020) China: Heavy-duty: fuel consumption. Transport Policy. https://www.transportpolicy.net/standard/china-heavy-duty-fuel-consumption/. Accessed 11 December 2020.

[CR34] Tsiamis DA & Castaldi MJ (2016) Determining accurate heating values of non-recycled plastics (NRP). Dissertation, University of New York

[CR35] USEPA (2016) Air emissions from MSW combustion facilities. United States Environmental Protection Agency. https://archive.epa.gov/epawaste/nonhaz/municipal/web/html/airem.html. Accessed 5 December 2020

[CR36] USEPA (2020) Basic information about landfill gas. United States Environmental Protection Agency. https://www.epa.gov/lmop/basic-information-about-landfill-gas. Accessed 17 December 2020

[CR37] Van Caneghem J, Van Acker K, De Greef J, Wauters G, Vandecasteele C (2019). Waste-to-energy is compatible and complementary with recycling in the circular economy. Clean Technol Environ Policy.

[CR38] Wang W, Themelis NJ, Sun K, Bourtsalas AC, Huang Q, Zhang Y, Wu Z (2019). Current influence of China’s ban on plastic waste imports. Waste Disposal & Sustainable Energy.

[CR39] Wee ST, Abas MA, Mohamed S, Chen GK, Zainal R (2017). Good governance in national solid waste management policy (NSWMP) implementation: a case study of Malaysia. AIP Conference Proceedings.

[CR40] Wheeler M (2020) How the F&B sector can address the issue of plastic pollution. Food and Beverage Industry News. https://foodmag.com.au/how-the-fb-sector-address-the-issue-of-plastic-pollution/. Accessed 22 December 2020

[CR41] Yong ZJ, Bashir MJ, Ng CA, Sethupathi S, Lim JW, Show PL (2019). Sustainable waste-to-energy development in Malaysia: appraisal of environmental, financial, and public issues related with energy recovery from municipal solid waste. Processes.

[CR42] Zainul E (2018) Worldwide Holdings to spend RM1b to develop waste-to-energy system in Selangor. The Edge Markets. https://www.theedgemarkets.com/article/worldwide-holdings-plans-rm1b-wastetoenergy-system-selangor#:~:text=SHAH%20ALAM%3A%20Worldwide%20Holdings%20Bhd,targeted%20for%20completion%20in%202024. Accessed 17 December 2020

